# Evaluation of 3D-CRT vs. VMAT for radiotherapy of whole breast with simultaneous integrated boost

**DOI:** 10.2340/1651-226X.2026.45677

**Published:** 2026-06-18

**Authors:** Gracinda Johansson, Rafat Kojoj, Sewa Surdashi, Martin Olin, Emil Fredén, Pelin Sen, Eija Dahl, Camilla Wendt

**Affiliations:** a Medical Physics, Diagnostics and Technology, Södersjukhuset, Stockholm, Sweden; b Department of Clinical Science and Education, Karolinska Institutet, Stockholm, Sweden; c Department of Oncology, Södersjukhuset, Stockholm, Sweden

**Keywords:** adjuvant radiotherapy, breast cancer, simultaneous integrated boost, volumetric modulated arc therapy, three-dimensional conformal radiotherapy

## Abstract

**Background and purpose:**

This study evaluates volumetric modulated arc therapy (VMAT) as a potential alternative to three-dimensional conformal radiotherapy (3D-CRT) for whole-breast radiotherapy (RT) with simultaneous-integrated boost (SIB).

**Patient/material and methods:**

Ten left-sided breast cancer patients previously treated in our institution (whole breast with SIB) were selected. Clinical plans were generated using tangential field-in-field 3D-CRT technique. VMAT plans were retrospectively created. Dosimetric evaluation was performed according to our clinical guidelines. A robustness evaluation of the 3D-CRT and VMAT plans against simulated anatomical and setup uncertainties was also performed. Differences between techniques were analysed using a two-sided Wilcoxon signed-rank test with a significance level of 0.05.

**Results:**

Both the 3D-CRT and VMAT plans fulfilled the clinical requirements for target volumes and organs at risk. VMAT plans showed a median increase in heart mean dose of 10.8%, compared to 3D-CRT (p < 0.05). Heart maximum dose was reduced by up to 56.7% with VMAT (*p* < 0.05), offering a clinically meaningful advantage. For the ipsilateral lung, no significant difference in mean dose was observed, V16Gy was significantly lower and V4Gy was significantly increased with VMAT. VMAT plans improved dose conformity to the boost planning target volume, (*p* < 0.05). Median monitor units (MU) values of 371 MU (359–466 MU) vs 927 MU (752–1018 MU) were obtained with 3D-CRT and VMAT. VMAT remained robust to simulated uncertainties for whole-breast but showed greater sensitivity in the boost volume.

**Interpretation:**

VMAT was a feasible alternative to 3D-CRT for whole-breast RT with SIB, improving boost dose-conformity and reducing maximum heart dose. However, VMAT requires careful dosimetric evaluation and robustness assessment.

## Introduction

Breast-conserving surgery followed by adjuvant whole-breast radiotherapy (RT) constitutes a cornerstone in the management of early-stage breast cancer, providing excellent local control and survival outcomes [1–3]. The addition of a tumour bed boost has been shown to further reduce the risk of local recurrence, particularly in younger patients and those with adverse pathological features [4–6]. Traditionally, boost irradiation has been delivered sequentially after completion of whole-breast RT [7]. In recent years, the simultaneous integrated boost (SIB) technique has gained increasing clinical interest, as it allows the delivery of differential dose levels to the whole-breast and tumour bed within a single treatment course, thereby reducing overall treatment time and potentially limiting unnecessary radiation exposure to surrounding organs at risk (OARs).

Three-dimensional conformal radiotherapy (3D-CRT) using tangential fields remains the standard technique for whole-breast RT in many institutions, owing to its robustness and favourable sparing of critical structures. The introduction of field-in-field (FiF) techniques has further improved dose homogeneity and reduced high-dose regions within the breast, contributing to improved treatment quality [8]. Nevertheless, when SIB is delivered using 3D-CRT, relatively large volumes of breast tissue may receive the higher boost dose, which has been associated with an increased risk of late normal tissue toxicity, including fibrosis [9]. Furthermore, achieving optimal dose conformity to the boost volume with 3D-CRT can be challenging, particularly in the presence of complex target geometries, potentially resulting in compromises between target coverage and normal tissue sparing.

Modern inverse-planning techniques such as volumetric modulated arc therapy (VMAT) offer the potential to improve plan quality by achieving superior dose conformity and sharper dose gradients around the boost volume [10]. However, the clinical implementation of VMAT in breast RT remains controversial. A major concern is the increased low-dose exposure to large volumes of normal tissue, which has been associated with a potential elevated risk of radiation-induced secondary malignancies [11]. The use of deep inspiration breath hold (DIBH) has been shown to significantly reduce cardiac dose by increasing the distance between the heart and the chest wall, thereby improving cardiac sparing across different RT techniques [12–15]. When combined with advanced delivery methods such as VMAT, DIBH may mitigate some of the disadvantages associated with increased low-dose exposure and heart dose.

Despite the increasing availability of VMAT and its demonstrated dosimetric advantages in selected scenarios, its role as an alternative to well-established tangential 3D-CRT for whole-breast RT with SIB remains institution-dependent. Comparative evaluations based on clinically implemented planning strategies are still limited [16, 17], and systematic dosimetric assessments are essential to support evidence-based clinical decision-making. The aim of this study was to evaluate VMAT as a potential alternative to clinical 3D-CRT for whole-breast RT with SIB in left-sided breast cancer patients treated in DIBH.

## Patients/material and methods

### Patients and structure delineation

Ten left-sided breast cancer patients previously treated with breast conserving surgery followed by whole-breast RT with SIB at Stockholm South Hospital (Stockholm, Sweden) were selected. Patients with boost volumes located centrally or medially in the breast were deemed pertinent for this study, since treatment plans with 3D-CRT result in large volumes of the breast receiving boost dose levels. The prescription dose was 2.67 Gy for the whole-breast and 3.20 Gy for the boost volume, given in 15 fractions to total dose of 40.05 and 48.0 Gy, respectively. Planning computed tomography (CT) images were acquired on a Siemens Somatom Definition Edge (Siemens Healthineers AG, Erlangen, Germany). A Wing Boards^TM^ (CIVCO Radiotherapy, Iowa, USA) was used to immobilise the patient in head-first, supine position with both arms above the head. The CT images consisted of 3-mm axial slices and were acquired in DIBH. DIBH was implemented using surface-guided radiation therapy (SGRT) with the Catalyst system (C-RAD AB, Uppsala, Sweden), which uses chest surface displacement as a surrogate for inspiration level. Patients were initially positioned in free breathing (FB) using a reference surface that was obtained with the Sentinel system (C-RAD AB, Uppsala, Sweden), during the planning-CT acquisition. From this reference surface, an individualised gating reference point was defined on the patient’s chest. A stable reference respiratory curve was also obtained. The gating window was set to 3-mm absolute vertical displacement of the gating point, with a DIBH lift of the reference gating point from baseline of typically 10 mm, individualised per patient. Residual variability in inspiration level is acknowledged as a source of geometric uncertainty and is reflected in the planning target volume (PTV) margins applied.

The target volumes were delineated by a radiation oncologist, following our clinical guidelines [18]. The clinical target volume (CTV) of the whole breast (CTV_L_40.05) included the whole breast, while the clinical target volume of the primary tumour (CTVT) for boost volume (CTVT_L_48.0) included the tumour bed and was delineated based on the surgical clips implanted during the breast conserving surgery. Patient characteristics including the volumes of the CTVT_L_48.0 and CTV_L_40.05 as well as the location of the boost volume in the breast are shown in Table 1.

**Table 1:** Patient and target volume characteristics.

Patient#	CTVT_L_48.0 volume (cm^3^)	CTV_L_40.05 volume (cm^3^)	Volume ratio (%)	Tumour bed location in the breast
1	24.66	383.75	6.43	Central
2	48.82	867.35	5.63	Central
3	25.38	385.62	6.58	Central
4	45.59	836.10	5.45	Medial
5	12.79	254.37	5.03	Medial
6	17.51	547.21	3.20	Central
7	39.11	683.16	5.73	Central
8	21.21	718.41	2.95	Central
9	45.45	479.47	9.48	Central
10	19.38	373.04	5.20	Central

For each patient, the volumes of the boost CTV (CTVT_L_48.0) and the whole-breast CTV (CTV_L_40.05) are reported together with the volume ratio (CTVT_L_48.0 / CTV_L_40.05) and the anatomical location of the tumour bed within the breast. CTV: clinical target volume.

The PTV for the whole breast and the boost volume were created by adding a uniform margin of 7 mm. Both the CTV and the PTV were cropped 5 mm under the body surface to exclude the build-up region of the photon depth-dose distribution in the dose statistics for the target volumes. The OAR were auto-generated by an atlas-based auto-segmentation (ABAS) software (Elekta AB, Stockholm, Sweden) and corrected by a radiation oncologist in Monaco treatment planning system (TPS) (Elekta AB, Stockholm, Sweden). The OARs considered were the heart, both lungs and the contralateral breast.

### Treatment planning

Clinical treatment plans were created in Monaco TPS using tangential FiF 3D-CRT technique. Treatment predominantly employed 6 MV photon beams, with 15 MV photons incorporated selectively as low-weight complementary fields when necessary to improve dose homogeneity and target coverage, particularly in patients with larger breast volumes. The plan isocentre was positioned in the centre of the PTV_L_40.05 longitudinally and vertically. In the lateral direction, the isocentre was placed at ca 8 cm from the midline. The incident beam angles for the medial and lateral tangential fields were optimised to maximise sparing of the heart and the ipsilateral lung. All fields were delivered with a collimator angle of 0°, and the multi-leaf collimator (MLC) was used to block the OARs. Additionally, the tangential fields were extended beyond the patient surface by approximately 2.5 cm to account for expected anatomical changes, such as breast swelling, as well as residual setup and positioning uncertainties affecting the areas close to the patient surface. Dose calculation of the clinical 3D-CRT plans was performed with the collapsed-cone algorithm (dose-to-medium and dose grid of 3 mm). To ensure comparability between techniques, all 3D-CRT plans were recalculated with Monte Carlo algorithm, consistent with the algorithm applied for VMAT planning (dose reported as dose-to-water, a dose grid of 3 mm and a statistical uncertainty of 1% per calculation).

**Figure 1:**
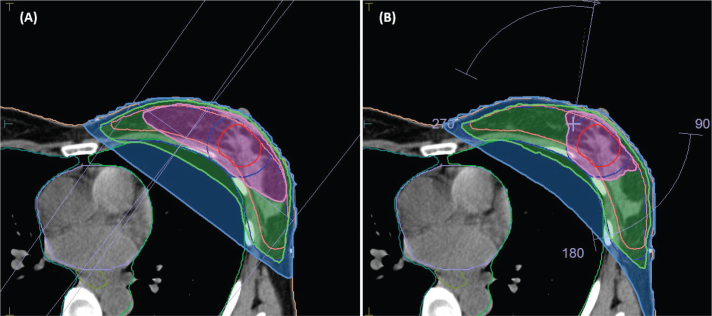
Dose distributions superimposed on a computed tomography (CT) slice shown in transversal view for treatment plans with 3D-CRT (A) and VMAT (B) technique. Dose levels shown are the 95% of the prescribed dose to the boost volume (pink area) and 95% of the prescribed dose to the breast volume (green area). The blue area represents the 20.025 Gy isodose level. The field arrangements used for the 3D-CRT and the VMAT plan are also shown. 3D-CRT: three-dimensional conformal radiotherapy; VMAT: volumetric modulated arc therapy.

For each patient, VMAT plans were retrospectively created using two tangential VMAT fields, each with three partial arcs of 75°. Photon beam energy was restricted to 6 MV. A collimator angle of 0° was used for all the VMAT fields. The field configuration for the VMAT plans is illustrated in Figure 1. The start angle of the medial VMAT field was adjusted for the individual patients to avoid the contralateral breast and to block the heart. The isocentre position was similar as for the 3D-CRT plans; however, vertically, the isocentre was placed closer to the patient surface. The dose calculation was made using the Monte Carlo algorithm, considering dose deposition in water, a dose grid of 3 mm and a statistical uncertainty of 1% per calculation. The maximum number of control points per arc was set to 90, and the minimum segment width allowed was 1 cm. A skin flash of 2.5 cm was used to improve the dose-coverage of the superficial parts of the breast in the presence of anatomical variation (e.g. breast swelling). A surface margin of 4 mm was applied to account for dose calculation and delivery limitations in the photon beam build-up region (in the patient surface). VMAT optimisation was made using the Pareto approach available in Monaco TPS, with manually assigned weights of the cost-functions for the target volumes and the OARs. All plans were optimised aiming to achieve a clinical acceptable dose coverage to the target volumes while maintaining dose to the OARs as low as possible, based on our clinical dose–volume (DV) criteria given in Table 2.

**Table 2:** Median values and range (minimum–maximum) of target and OAR dose–volume metrics for the clinical 3D-CRT plans and the corresponding VMAT plans.

Structure	DV-metric	3D-CRT	VMAT
Heart	*D*mean < 4 Gy	1.41 (0.98–2.45)	1.57 (1.24–2.23)
	D_0.03cm₃_ < 51.84 Gy	30.1 (6.13–38.25)	16.16 (6.55–28.36)*
Lung_L	*D*mean < 8 Gy	5.46 (2.81–8.42)	5.31 (3.66–7.75)
	V16Gy < 20%	11.67 (4.51–19.87)	8.14 (2.69–15.58)*
	V4Gy < 55%^†^	23.44 (3.30–35.31)	38.48 (28.9–50.13)*
Breast_R	*D*mean < 2.5 Gy	0.67 (0.44–0.79)	1.69 (1.31–2.33)*
Lung_R	*D*mean < 2 Gy	0.38 (0.29–0.45)	0.74 (0.66–1.00)*
PTV_L_40.05	D98% > 37.25 Gy	37.74 (37.21–38.64)	37.41 (37.28–38.08)
	V45.6Gy < 40%	26.52 (22.46–32.3)	12.04 (7.78–17.42)*
CTV_L_40.05	D98% > 38.05 Gy	38.65 (38.22–39.04)	39.60 (38.74–39.78)
PTVT_L_48.0	D98% > 44.64 Gy	45.49 (43.45–46.18)	45.27 (44.65–46.03)
	CI = V95%/PTV	2.94 (1.69–4.53)	1.23 (1.11–1.43)*
CTVT_L_48.0	*D*mean > 48.0 Gy	48.81 (48.04–49.67)	48.86 (48.68–49.13)
	*D*min > 45.6 Gy	46.54 (45.64–47.60)	46.39 (45.61–47.08)

* *P* < 0.05 statistically significant; otherwise, not significant.

† Taken from the Radiation Therapy Oncology Group (RTOG) 1005 protocol. OAR: organs at risk; 3D-CRT: three-dimensional conformal radiotherapy; VMAT: volumetric modulated arc therapy; DV: dose–volume; CTV: clinical target volume; PTV: planning target volume.

### Plan evaluation

A comparison between the clinical 3D-CRT plans and the VMAT plans was made by means of evaluation of the relevant DV-metrics for the target volumes and the OARs, according to our clinical guidelines (Table 2), which are based on the Swedish national guidelines for breast cancer RT [18]. The conformity index (CI) [19] for the boost volume, defined as the ratio of the volume of the 95% isodose and the volume of the boost Planning target volume of the primary tumor (PTVT)_L_48.0 was also evaluated.

### Robustness analysis

The robustness of the 3D-CRT and VMAT plans was evaluated by simulating clinically relevant anatomical and setup uncertainties. Anatomical changes due to breast swelling were modelled by recalculating the original treatment plans on modified CT datasets in which a uniform virtual bolus of 5, 10 or 15 mm thickness was applied to the external breast contour, without modifying the target volumes or replanning. The bolus was assigned the electron density of water to realistically represent soft tissue swelling. This approach enabled a systematic assessment of the dosimetric impact of progressive breast enlargement on target dose coverage on the original plan, representing a conservative worst-case scenario for dose-coverage degradation. Setup uncertainties were investigated by simulating patient positioning errors through isocentre shifts applied in six directions. A translational displacement of 7 mm was used, corresponding to the clinically used PTV margin. This simulation reflects worst-case residual setup errors after patient positioning and image guidance.

For each simulated scenario, DV metrics were extracted to quantify plan robustness in terms of target dose-coverage. Specifically, D98% for the CTV_L_40.05 and minimum and mean dose (*D_min_* and *D_mean_*) for the CTVT_48.0 were recorded for all generated treatment plans.

### Treatment plan verification

Treatment delivery was performed using an Elekta Versa HD (Elekta AB, Stockholm, Sweden) linear accelerator. For both the 3D-CRT and the VMAT plans, the resulting total monitor units (MU) per plan and the total beam-on time were recorded. Both the 3D-CRT and the VMAT plans were verified using the Delta^4^ (ScandiDos AB, Uppsala, Sweden) phantom. A gamma index evaluation with a distance-to-agreement of 3 mm and a percentage dose difference of 3% was performed. The lower threshold of gamma passing rate (GPR) was set to 95%, according to our clinical routine.

### Statistical analysis

A two-sided Wilcoxon signed-rank test with a significance level of 0.05 was performed to analyse the differences in paired DV metrics obtained with the clinical 3D-CRT and the VMAT plans.

## Results

### Plan evaluation

**Figure 2:**
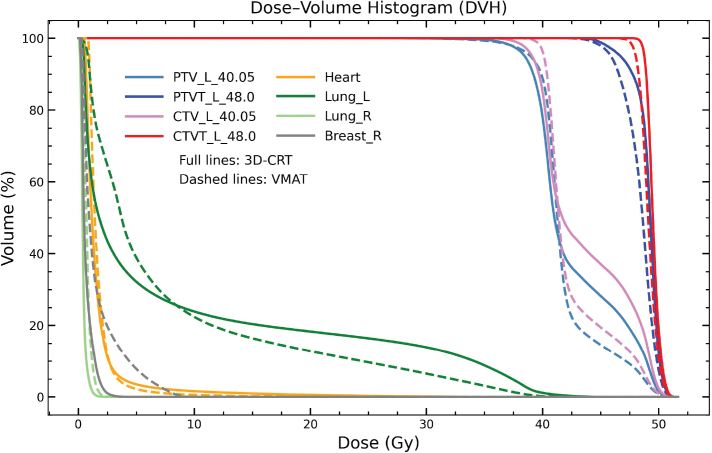
Dose–volume histogram (DVH) comparison between clinical 3D-CRT plan (full lines) and VMAT (dashed lines) for a representative patient. 3D-CRT: three-dimensional conformal radiotherapy; VMAT: volumetric modulated arc therapy; CTV: clinical target volume; PTV: planning target volume.

Typical dose ditributions obtained with 3D-CRT and VMAT are shown in Figure 1 and the dose-volume histogram (DVH) comparison is presented in Figure 2. Both the 3D-CRT and VMAT plans fulfilled the predefined clinical requirements for target coverage and OAR dose constraints. Compared with 3D-CRT, VMAT was associated with a median increase in mean heart dose of 10.8% (range -9.1 to 26.7%, *p* < 0.05). In contrast, the maximum heart dose was reduced by up to 56.7% with VMAT (*p* < 0.05). For the ipsilateral lung, no significant difference in mean lung dose was observed (median 5.46 Gy with 3D-CRT vs. 5.31 Gy with VMAT, *p* > 0.05), whereas V16Gy was significantly lower with VMAT (8.14%) compared to 3D-CRT (11.67%), *p* < 0.05. The V4Gy of the ipsilateral lung was consistently higher with VMAT (range, 28.9–50.1%) compared to 3D-CRT (range, 3.3–35.3%), *p* < 0.05. For the contralateral breast and the contralateral lung, significantly higher mean dose was registered with VMAT (*p* < 0.05); however, the registered values were below our clinical tolerance levels for these two OARs (Table 2).

VMAT provided significantly improved dose conformity to PTVT_L_48.0 compared with 3D-CRT (*p* < 0.05). The median CI was 1.23 (range 1.11–1.43) for VMAT and 2.94 (range 1.69–4.53) for 3D-CRT.

### Robustness evaluation

With the 5-mm simulated swelling, the CTV_L_40.05 D98% criterion (D98% > 38.05 Gy) was fulfilled for seven patients with 3D-CRT and for all patients with VMAT. For CTVT_48.0, the *D*_mean_ criterion (*D*_mean_ > 48.0 Gy) was met in 30% (3D-CRT) and 70% (VMAT), while the *D*_min_ criterion (*D*_min_ > 45.6 Gy) was fulfilled in 50 and 70%, respectively.

With 10-mm swelling, the CTV_L_40.05 D98% criterion was met in 0% (3D-CRT) and in 90% (VMAT) of the plans. For CTVT_48.0, *D*_mean_ was fulfilled in 0% (3DCRT) and 10% (VMAT). CTVT_48.0 *D*_min_ was met in 20% (3DCRT) vs 10% (VMAT).

With the 15-mm swelling, CTV_L_40.05 D98% criterion was not fulfilled in any of the cases with 3D-CRT or VMAT plans. For CTVT_48.0, both *D*_mean_ and *D*_min_ criteria were met in one VMAT case and in none of the 3D-CRT plans.

Under simulated ±7-mm isocentre shifts in the three translational directions, the CTV_L_40.05 D98% criterion was met in 83% of all simulated scenarios with 3D-CRT and 93% with VMAT. For CTVT_48.0, the *D*_mean_ criterion was met in 88 and 87% and the *D*_min_ criterion was fulfilled in 37 and 15%, for 3D-CRT and VMAT, respectively.

**Figure 3:**
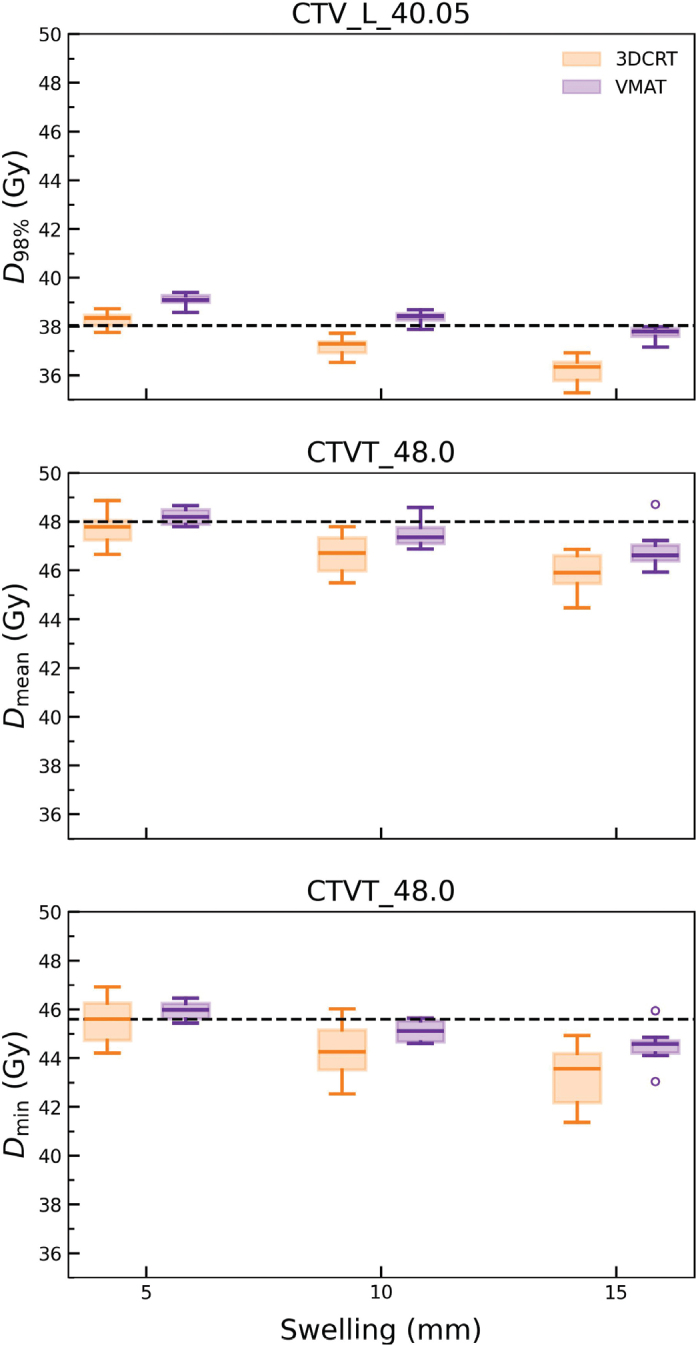
Boxplot showing the dose–volume metric for the CTV_L_40.05 and CTVT_L_48.0 depicting the results of the robustness evaluations of the simulation of breast swelling with uniform thickness of 5, 10 and 15 mm for 3D-CRT (orange) and VMAT (purple). 3D-CRT: three-dimensional conformal radiotherapy; VMAT: volumetric modulated arc therapy; CTV: clinical target volume.

**Figure 4:**
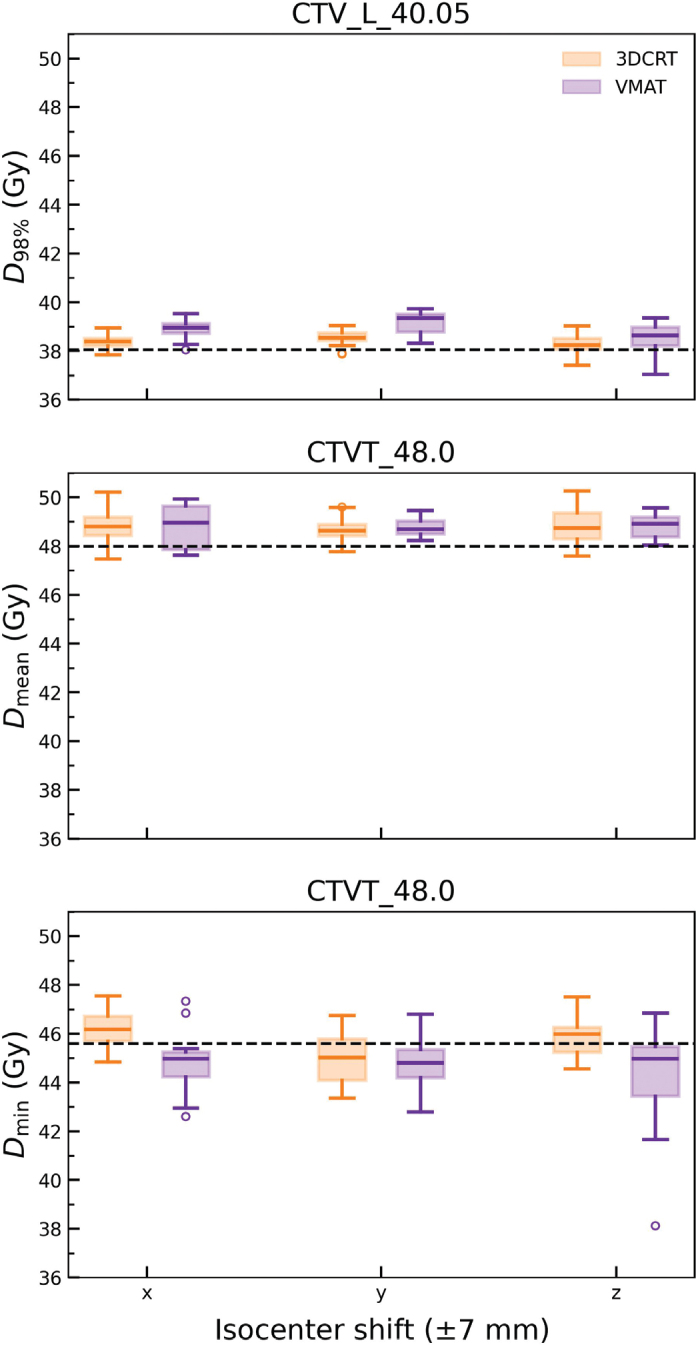
Boxplot illustrating dose–volume metrics for the CTV_L_40.05 and CTVT_L_48.0 depicting the results of the robustness evaluation of the simulated setup errors of 7 mm in three translational axis, for 3D-CRT (orange) and VMAT (purple). 3D-CRT: three-dimensional conformal radiotherapy; VMAT: volumetric modulated arc therapy; CTV: clinical target volume.

**Figure 5:**
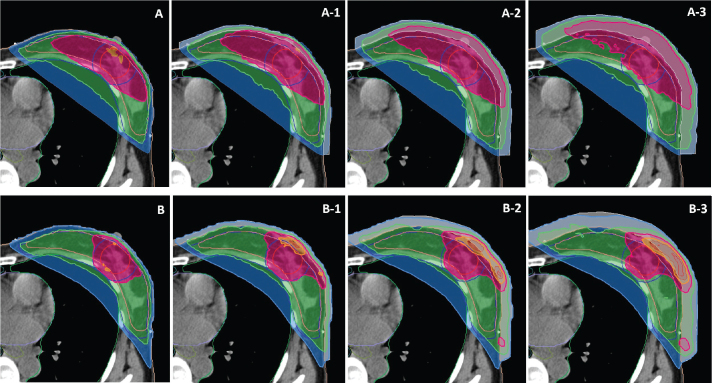
Illustration of dose degradation in the axial view for 3D-CRT (upper panels) and VMAT (lower panels) with the introduction of bolus of 5 mm (A-1, B-1), 10 mm (A-2, B-2) and 15 mm (A-3, B-3). The original plans are shown in A (3D-CRT) and B (VMAT). 3D-CRT: three-dimensional conformal radiotherapy; VMAT: volumetric modulated arc therapy.

A summary of the robustness evaluation is illustrated in Figures 3, 4 and 5.

### Treatment plan verification

Median MU per plan were 371 MU (range 359–466 MU) for 3D-CRT and 927 MU (range 752–1018 MU) for VMAT. Beam-on time ranged from 57 to 109 seconds (0.95–1.82 minutes) for 3D-CRT and from 155 to 177 seconds (2.58–2.95 minutes) for VMAT. Both the VMAT and the 3D-CRT plans met our clinical tolerance values for the PSQA, with a GPR of at least 95.4% (3D-CRT) and 98.6% (VMAT), Table 3.

**Table 3:** Total monitor units (MU) per plan, beam-on time and results for patient-specific quality assurance (PSQA) performed for the 3D-CRT and the VMAT plans.

Patient#	MU		Beam-on time (s)		Gamma index 3 mm/3% (%)	
	3D-CRT	VMAT	3D-CRT	VMAT	3D-CRT	VMAT
1	359	932	67.4	176.0	98.9	99.9
2	373	1003	75.7	174.0	97.8	100.0
3	366	902	72.8	177.0	96.9	100.0
4	392	910	81.0	169.0	96.5	100.0
5	359	955	57.1	166.0	99.9	100.0
6	371	988	70.5	172.0	97.6	100.0
7	385	921	71.8	161.0	98.5	100.0
8	446	1018	108.5	155.0	95.4	100.0
9	370	862	69.6	161.0	97.9	98.6
10	359	752	64.0	173.0	99.9	99.0

3D-CRT: three-dimensional conformal radiotherapy; VMAT: volumetric modulated arc therapy; MU: monitor units.

## Discussion and conclusion

In this study, VMAT was evaluated as a potential alternative to tangential FiF 3D-CRT for left-sided whole-breast RT with SIB in patients treated with DIBH. Both techniques fulfilled institutional planning objectives for target coverage and OAR constraints. However, differences were observed in dose conformity to the boost volume, heart dose, plan robustness and treatment efficiency.

A key finding was the significantly improved dose conformity to the boost PTV achieved with VMAT. This finding is consistent with previous studies demonstrating that inverse-planned techniques generate steeper dose gradients and better conform to complex target geometries [20, 21]. Improved conformity is particularly relevant in SIB treatments, where excessive high-dose exposure of surrounding breast tissue has been associated with increased risk of late toxicity, including fibrosis [4, 5]. The European Organisation for Research and Treatment of Cancer (EORTC) boost trial demonstrated higher rates of breast fibrosis in patients receiving boost irradiation, highlighting the importance of limiting unnecessary high-dose exposure [5, 22]. Although clinical outcomes were not assessed in the present study, the reduced high dose spread observed with VMAT may have implications for long-term cosmetic results.

With respect to heart dose, VMAT resulted in a modest but statistically significant increase in mean heart dose compared with 3D-CRT, while significantly reducing maximum heart dose. This reflects the fundamental difference between conventional and intensity-modulated techniques. 3D-CRT limits low-dose spread outside the treatment fields, whereas VMAT redistributes dose more uniformly, reducing focal hotspots at the expense of a broader low-dose bath. Importantly, in the present study, the mean heart dose remained considerably lower than our clinical constraints with both techniques (*D*_mean_ < 4 Gy). Epidemiologic evidence indicates a relationship between mean heart dose and long-term cardiac risk [13, 23]. The clinical relevance of maximum dose to small cardiac sub-volumes remains less clearly defined [24]. Increasing evidence suggests that mean heart dose alone may not fully capture cardiac risk, as high doses to cardiac substructures such as the left anterior descending artery (LAD) may occur even when global heart doses remain low [25–27]. Wennstig et al. [28] suggested in their retrospective study on breast cancer RT, the inclusion of LAD dose in breast RT planning, with a clear correlation of LAD dose with the risk of radiation-induced stenosis.

The use of DIBH represents an essential component of this treatment approach. DIBH is well established as an effective strategy to reduce heart dose in left-sided breast cancer [12, 13, 29] and likely reduced absolute cardiac exposure for both techniques. The combination of DIBH with conformal techniques such as VMAT may allow improved boost conformity while maintaining acceptable heart sparing, if robustness considerations are addressed.

For the ipsilateral lung, VMAT reduced V16Gy without significantly improving the mean lung dose. The reduction of these DV metrics has also been reported in other studies of VMAT technique for breast cancer RT [17]. Mean lung dose and intermediate DV metrics to the lung (e.g. V15Gy–V20Gy) have been correlated to the risk of radiation-induced pneumonitis following RT [30–33]. Therefore, the relatively lower V16Gy with VMAT could be expected to reduce the incidence of radiation-induced pneumonitis for this patient group. VMAT was also associated with a statistically significant increase in V4Gy for the ipsilateral lung (Table 2), consistent with the low-dose spread inherent to rotational delivery techniques [20, 34]. Nevertheless, all V4Gy values registered for the VMAT plans remained within the constraint used in the Radiation Therapy Oncology Group (RTOG) 1005 study protocol (V4Gy < 55%) [35, 36]. The toxicity implications arising from the low dose lung irradiation were not evaluated in the present study. However, it warrants consideration in patients with pre-existing pulmonary comorbidities, concurrent pulmonary-toxic systemic therapies, or anticipated re-irradiation. When clinically indicated, stricter ipsilateral lung objectives can effectively reduce V4Gy through constraint reweighting during VMAT optimisation, provided target coverage is not compromised.

Plan robustness represents a central concern when SIB is delivered using highly conformal techniques. Tangential 3D-CRT is traditionally regarded as geometrically robust due to its relatively homogeneous fluence distribution and broader high-dose regions [37]. In contrast, VMAT achieves improved conformity through increased modulation and steeper dose gradients, which may increase sensitivity to geometric perturbations [38, 39]. In the robustness analysis carried out in the present study, CTV_L_40.05 D98% was preserved for both techniques under 5-mm swelling and ± 7-mm isocentre shifts. At 10-mm swelling, VMAT maintained dose-coverage to the whole-breast CTV more frequently than 3D-CRT (90% vs 40%), suggesting improved resilience to moderate anatomical expansion. However, at 15-mm swelling, marked deterioration was observed for both techniques (Figure 3). For the boost volume (CTVT_L_48.0), robustness was lower overall. With increasing swelling, both *D*_mean_ and particularly *D*_min_ declined for both techniques, without a consistent advantage for VMAT at ≥ 10 mm (Figure 3). Under simulated ± 7-mm shifts (Figure 4), boost *D*_min_ was fulfilled in 62% of scenarios with 3D-CRT compared with 15% with VMAT. This indicates greater sensitivity of the more conformal VMAT dose distribution to translational setup uncertainties. These findings suggest that improved conformity does not necessarily translate into improved geometric robustness [40], particularly for small high-dose boost volumes.

Treatment efficiency also differed between the evaluated treatment plans (Table 3). While both 3D-CRT and VMAT fulfilled our clinical requirement for PSQA, VMAT required substantially higher MUs and approximately twice the beam-on time compared with 3D-CRT. Beam-on time ranged from 155 to 177 seconds for VMAT and 57–109 seconds for 3D-CRT. In DIBH treatments, longer beam-on time necessitates prolonged or repeated breath-holds, which may increase intra-fraction variability [41, 42]. Although DIBH reproducibility is generally high [29], small inter- and intra-breath-hold variations have been reported [43, 44] and may affect dose delivery, particularly for steep dose gradients. The increased sensitivity of boost *D*_min_ observed with VMAT may therefore reflect the combined effect of conformal dose distribution and residual geometric variability during repeated breath-holds. Careful breath-hold coaching and motion monitoring are therefore important when VMAT is delivered in DIBH.

This study has a few limitations. The cohort size was limited, and the analysis was restricted to dosimetric parameters, which precludes any conclusions regarding clinical outcomes or treatment-related toxicity. Additionally, only a single VMAT planning strategy was evaluated; alternative arc configurations or hybrid planning approaches may exhibit different robustness characteristics [16, 17, 45, 46]. The analysis reflects routine clinical planning practice and provides relevant information to support institutional decision-making. Furthermore, the robustness analysis for breast swelling was performed by applying a virtual bolus to the planning CT and recalculating dose on the original plan (Figure 5), without extending the CTV or PTV into the swollen volume. While this approach is consistent with published methodology [47, 48] and represents a clinically relevant worst-case scenario, it likely overestimates true coverage in practice.

In conclusion, VMAT fulfilled all predefined planning objectives for whole-breast RT with SIB and provided significantly improved dose-conformity to the boost volume compared with tangential 3D-CRT. VMAT reduced maximum heart dose but was associated with a modest increase in mean heart dose. In the robustness analysis, dose-coverage to whole breast was preserved for both techniques under moderate anatomical changes and setup shifts, whereas the minimum dose given to the boost volume demonstrated greater sensitivity, particularly for VMAT under translational shifts. When combined with DIBH, VMAT represents a feasible alternative to 3D-CRT for selected patients, provided that the trade-off between improved conformity and reduced geometric robustness is carefully considered.

## Data Availability

The data supporting the findings of this study are available from the corresponding author (GJ) upon request.
